# Immune response enhancement by dietary supplementation with *Caesalpinia sappan* extract in weaned pigs challenged with porcine reproductive and respiratory syndrome virus

**DOI:** 10.1186/s12917-024-03911-5

**Published:** 2024-03-22

**Authors:** Chaiwat Arjin, Surat Hongsibsong, Kidsadagon Pringproa, Warintorn Ruksiriwanich, Chompunut Lumsangkul, Jirapat Arunorat, Phongsakorn Chuammitri, Mintra Seel-audom, Sarana Rose Sommano, Korawan Sringarm

**Affiliations:** 1 Department of Animal and Aquatic Sciences, Faculty of Agriculture, Chiang Mai University, Chiang Mai, 50200 Thailand; 2 School of Health Science Research, Research Institute for Health Sciences, Chiang Mai University, Chiang Mai, 50200 Thailand; 3 Department of Veterinary Bioscience and Veterinary Public Health, Faculty of Veterinary Medicine, Chiang Mai University, Chiang Mai, 50200 Thailand; 4 Cluster of Agro Bio-Circular-Green Industry (Agro BCG), Chiang Mai University, Chiang Mai, 50200 Thailand; 5 Department of Pharmaceutical Sciences, Faculty of Pharmacy, Chiang Mai University, Chiang Mai, 50200 Thailand; 6 Department of Plant and Soil Sciences, Faculty of Agriculture, Chiang Mai University, Chiang Mai, 50200 Thailand

**Keywords:** Porcine reproductive and respiratory syndrome virus, Plant extract, *Caesalpinia sappan*, Productive performance, Antibody titer

## Abstract

**Background:**

At present, porcine reproductive and respiratory syndrome (PRRS) caused by the PRRS virus (PRRSV) is one of the most severe epidemics impacting pig farming globally. Despite the fact that a number of studies have been conducted on potential solutions to this problem, none have proven effective. The focus of problem solving is the use of natural ingredients such as plant extracts. Popular throughout Asia, *Caesalpinia sappan* (CS) is a therapeutic plant that inhibits PRRSV in vitro. Therefore, this study was performed to determine the efficacy of CS extract dietary supplementation on the productive performance, antibody levels, immunological indicators, and lung pathology of PRRSV-challenged weaned pigs. A total of 32 weaned piglets (28 days old) were randomized into 4 groups and kept separately for 14 days. The treatments were organized in a 2 × 2 factorial design involving two factors: PRRSV challenge and supplementation with 1 mg/kg CS extract. The pigs in the PRRSV-challenged groups were intranasally inoculated with 2 mL of PRRSV (VR2332) containing 10^4^ TCID_50_/mL, while those in the groups not challenged with PRRSV were inoculated with 2 mL of normal saline.

**Results:**

In the PRRSV-challenged group (CS + PRRSV), supplementation with CS extract led to an increase in white blood cells (WBCs) on Day 7 post infection (*p* < 0.05) and particularly in lymphocytes on Days 7 and 14. The antibody titer was significantly greater in the CS + PRRSV group than in the PRRSV-challenged group not administered CS (PRRSV group) on Day 14 postinfection (S/P = 1.19 vs. 0.78). In addition, CS extract administration decreased the prevalence of pulmonary lesions, which were more prevalent in the PRRSV-challenged pigs that did not receive the CS extract.

**Conclusion:**

The findings of this study suggest that supplementation with CS extract is beneficial for increasing WBC counts, especially lymphocytes, increasing the levels of antibodies and reducing the prevalence of lung lesions in PRRSV-infected pigs.

**Supplementary Information:**

The online version contains supplementary material available at 10.1186/s12917-024-03911-5.

## Background

Porcine reproductive and respiratory syndrome (PRRS) has been one of the most economically significant swine diseases worldwide for more than two decades [1]. The disease is caused by the porcine reproductive and respiratory syndrome virus (PRRSV), which initially emerged in swine populations in Europe (type I) and the US (type II) in the early 1990s [2, 3]. PRRSV is a small single-stranded nonsegmented RNA virus with the virion in an envelope; it is spherical in shape and ranges from 45 to 80 nm in diameter [4]. PRRSV belongs to the *Arteriviridae* family (order *Nidovirales*), which is the same family as the equine arteritis, mouse lactate dehydrogenase-elevating, and simian hemorrhagic fever viruses [1]. PRRSV causes severe reproductive failure in sows, which leads to late-term abortion and stillbirth. It also induces respiratory disorders in piglets, which are associated with the porcine respiratory disease complex in combination with secondary infections [5]. However, as PRRS is caused by a viral infection, it is currently untreatable, and no drugs exist to cure or control it. The most effective method of controlling PRRSV infection is vaccination. However, vaccination has limitations in preventing this disease, such as the ability of live vaccines to mutate and cause PRRSV infections in pigs. Additionally, the cost of vaccination is high and increasing [6]. One possible option is the use of antibiotics to control secondary infections. Nonetheless, the use of antibiotics in animal production could lead to an increase in bacterial resistance in humans and animals, particularly regarding resistance to gram-negative bacteria, as well as an increase in production. In 1996, the mild form of PRRSV was first isolated from piglets with chronic respiratory distress in Thailand, and the virus was subsequently identified as the US genotype [7, 8]. Surveillance conducted annually from 2008 to 2013 revealed that both types I and II of PRRSV were circulating in Thai swine, with genotype II being more prevalent than genotype I. The prevalence of PRRSV in Thailand's pig populations was 13.86%, and genotype II was more prevalent than genotype I [9].

Consequently, many researchers have proposed a new strategy to solve these problems, focusing on using natural plant extracts that contain active ingredients that have antiviral properties. This alternative is considered safe, especially as it does not involve the administration of drugs, and does not lead to antibiotic resistance. Previously, *Cryptoporus volvatus* extract was found to exhibit antagonistic effects against PRRSV infection and replication [10]. Additionally, crude *Cynodon dactylon* extract significantly inhibited PRRSV replication as early as 24 h post infection [11]. Therefore, it is interesting to investigate the potential antiviral activity of plant extracts against PRRSV. Our previous studies revealed that *Caesalpinia sappan* (CS) crude extract has antiviral activity against PRRSV and inhibits PRRSV replication in vitro in the MARC-145 cell line [12]. In addition, semipurification of CS into six fractions revealed that the first fraction exhibited potential antiviral activity and inhibited PRRSV replication in a MARC-145 monolayer at 72 h postinfection (virus titer 2.75 median tissue culture infective dose(TCID_50_)/mL (log10) vs. 9.50 median log10 TCID_50_ in the control). This fraction was subsequently found to contain byakangelicin, brazilin, and naringenin [13]. CS is a plant in the Leguminosae family. It is a generally well-known medicinal plant that is distributed and planted in tropical Asian countries including southern China, India, Myanmar, and Thailand. The dried heartwood of CS has been utilized for centuries in oriental medicines, including Ayurveda and Traditional Chinese Medicine [14, 15]. Numerous biological features of CS, including its antioxidant [12, 14, 16], antibacterial [17, 18], anti-inflammatory [18, 19], and antiviral [12, 13, 20] properties, have been documented. However, there are no data available regarding the effectiveness of CS for preventing PRRSV infection in vivo. Therefore, the purpose of this study was to investigate the potential application of CS extract dietary supplementation for improving the productive performance of PRRSV-challenged weaned pigs. We also aimed to determine the efficacy of CS extract with regard to improving antibody levels, immunological indicators, and lung pathology in weaned pigs exposed to PRRSV.

## Results

### Productive performance

The productive performance of the pigs is shown in Table 1. The body weight of the pigs was equal among the groups before the experiment started (average body weight 9.15 ± 0.52 kg). The final weights of the pigs in all the treatment groups were not significantly different (*p* > 0.05). Moreover, there was no significant effect of CS extract supplementation, PRRSV challenge, or the interaction between CS extract supplementation and PRRSV challenge on the average daily feed intake (ADFI), weight gain (WG), the average daily gain (ADG), or the feed conversion ratio (FCR) throughout the experimental period (*p* > 0.05).

**Table 1:** Effect of supplementation of CS extract on productive performance of pigs with or without PRRSV challenge

	**No PRRSV**		**PRRSV**		**SEM**	***p*-value**		
	**- CS**	** + CS**	**- CS**	** + CS**		**PRRSV**	**CS**	**PRRSV x CS**
**Body weight**								
D0	9.20	9.13	9.19	9.10	0.188	0.950	0.825	0.976
D7	12.19	12.11	12.02	12.05	0.239	0.811	0.958	0.914
D14	14.67	14.65	14.10	14.33	0.192	0.259	0.781	0.744
**D 0–7**								
ADFI	0.46	0.47	0.45	0.46	0.003	0.201	0.066	0.618
WG	2.99	2.98	2.83	2.95	0.113	0.687	0.799	0.782
ADG	0.43	0.43	0.40	0.42	0.016	0.658	0.772	0.772
FCR	1.14	1.14	1.18	1.15	0048	0.815	0.876	0.825
**D 7–14**								
ADFI	0.83	0.83	0.81	0.85	0.013	0.852	0.575	0.429
WG	2.48	2.54	2.08	2.28	0.150	0.288	0.661	0.806
ADG	0.35	0.36	0.27	0.32	0.021	0.281	0.674	0.805
FCR	2.66	2.69	2.90	2.70	0.151	0.672	0.786	0.696
**D 0–14**								
ADFI	0.63	0.65	0.63	0.65	0.009	0.717	0.251	0.828
WG	5.47	5.52	4.91	5.24	0160	0.202	0.554	0.670
ADG	0.39	0.39	0.35	0.37	0.011	0.207	0.568	0.683
FCR	1.64	1.69	1.85	1.78	0.053	0.172	0.930	0.587

*No PRRSV* unchallenged PRRSV, *PRRSV* porcine reproductive and respiratory syndrome virus challenged, *CS* C. sappan extract, *WG* weight gain, *ADFI* average daily feed intake, *ADG* average daily gain, *FCR* feed conversion ratio

### Clinical signs

**Fig. 1:**
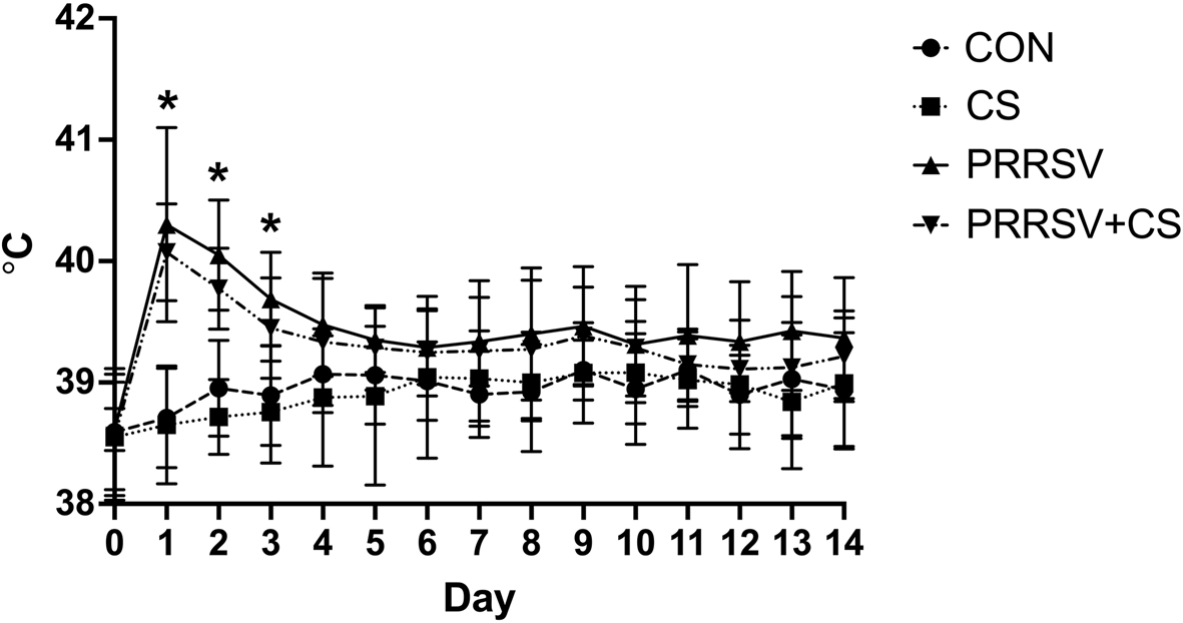
Effect of supplementation with CS extract on the rectal temperature of pigs with or without PRRSV challenge. The data are shown as the mean ± SD. The asterisks indicate significant differences (* *p* < 0.05; ** *p* < 0.01; *** *p* < 0.001; **** *p* < 0.0001) between groups

**Fig. 2:**
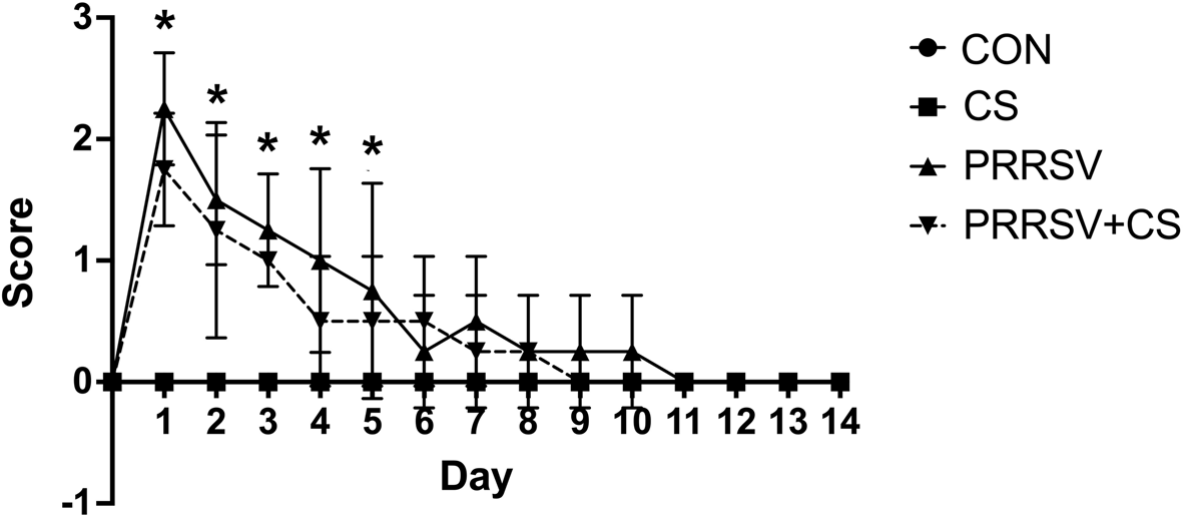
Effect of supplementation with CS extract on the clinical signs in pigs with or without PRRSV challenge. The data are shown as the mean ± SD. The asterisks indicate significant differences (* *p* < 0.05; ** *p* < 0.01; *** *p* < 0.001; **** *p* < 0.0001) between groups

During the first three days after being challenged with the virus, the PRRSV-challenged pigs (PRRSV and PRRSV + CS) exhibited significantly greater rectal temperatures than did the control pigs (*p* < 0.05) (Fig. 1). Nonetheless, on day four postinfection, the rectal temperature of the PRRSV-challenged pigs had decreased to the same level as that of the control pigs. In terms of clinical symptoms, all the pigs exhibited lethargy and anorexia following PRRSV inoculation during the initial two days of infection (Fig. 2). Additionally, dyspnea was found within the first two days after infection, but respiratory symptoms such as coughing were not observed. In contrast, no clinical symptoms were detected in the pigs that received a sham injection.

### Viremia

**Fig. 3:**
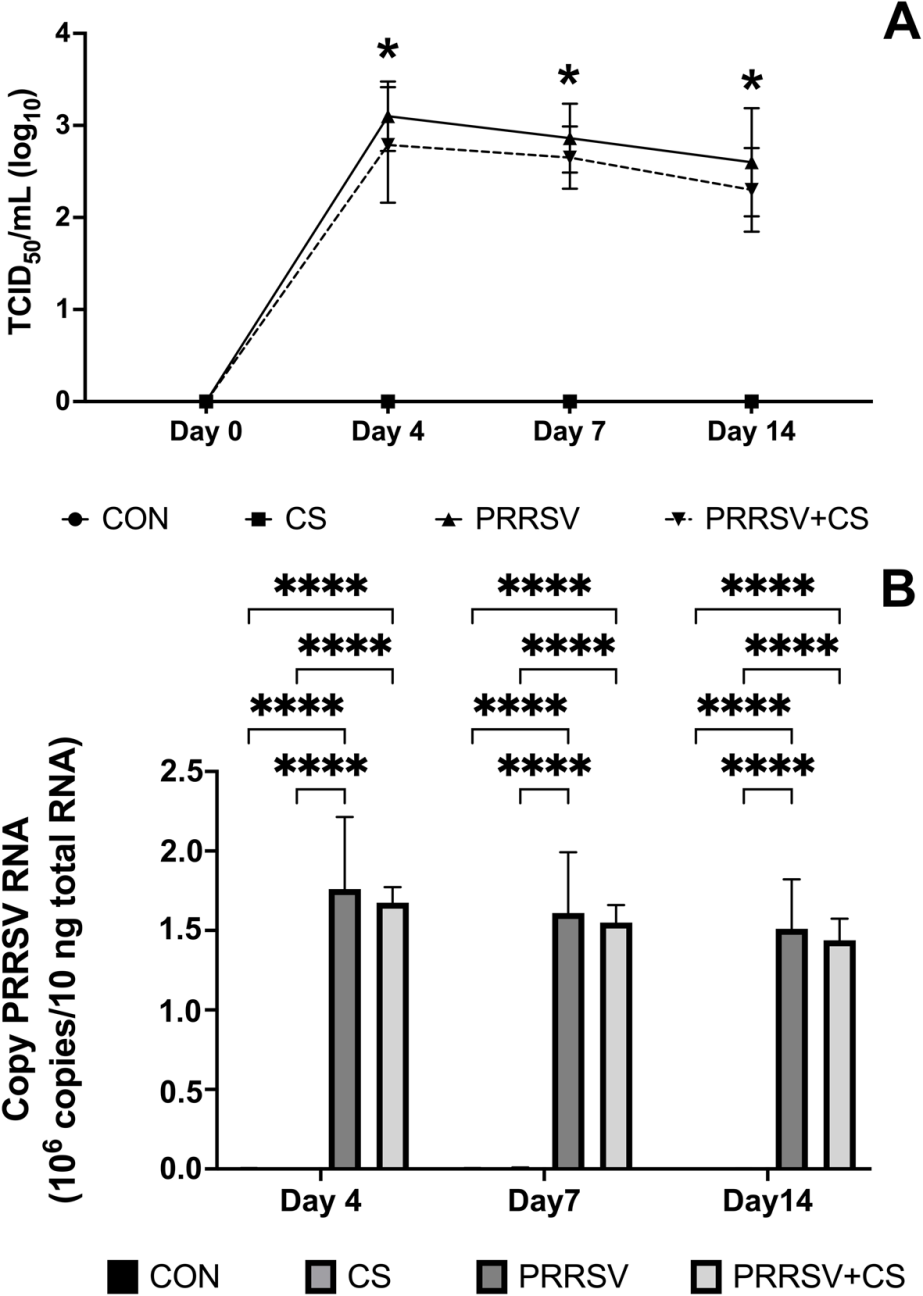
Effect of supplementation with CS extract on viraemia (**A**) and the number of PRRSV RNA copies (**B**) in the serum following PRRSV challenge. The data are shown as the mean ± SD. Asterisks indicate significant differences (* *p* < 0.05 and **** *p* < 0.0001) between groups

The viremia in the serum of the PRRSV-challenged pigs was determined using an IPMA assay and is expressed as the TCID_50_ log_10_/mL (Fig. 3A). After PRRSV was inoculated into the pigs, the viral load in the serum dramatically increased to 3.10 TCID_50_ log_10_/mL in the PRRSV-challenged group. However, the viral load in the serum of PRRSV-challenged pigs supplemented with CS extract was lower, at 2.79 TCID_50_ log_10_/mL. On Day 7 of the experiment, the viral load in the serum of the challenged pigs had slightly decreased, and it reached 2.86 and 2.6 TCID_50_ log_10_/mL on Day 14. Moreover, via RT‒qPCR, we detected that the degree of viremia in pig serum exhibited the same pattern as that observed via IPMA (Fig. 3B). On Day 4, a high copy number of PRRSV was found in the serum. It gradually decreased on Day 7 and Day 14 PI, but the difference was not significantly different.

### Antibody titer

**Fig. 4:**
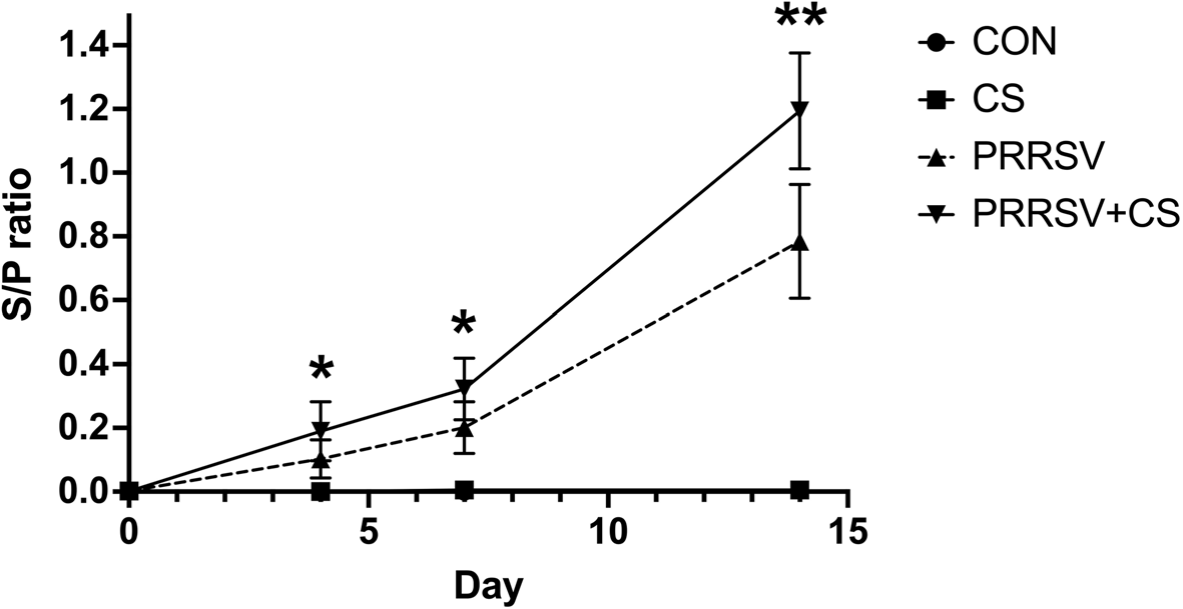
Effect of supplementation with CS extract on the antibody titer in pigs following PRRSV challenge. The data are shown as the mean ± SD. Asterisks indicate significant differences (* *p* < 0.05; ** *p* < 0.01) between groups

Figure 4 shows the PRRSV antibody titer in the serum of the pigs. The investigation revealed that both the PRRSV group and the CS + PRRSV group had S/P ratios less than 0.4 on Days 4 and 7 postinfection. On Day 14 postinfection, a PRRSV-specific antibody titer that was higher than 0.4 indicated positive detection. Compared with the PRRSV group (S/P = 0.78), the CS + PRRSV group had significantly (*p* < 0.0001) greater levels of PRRSV-specific antibodies (S/P = 1.19).

### Hematology

Red blood cell (RBC) count, hemoglobin (HGB), Hematocrit (HCT), mean corpuscular volume (MCV), mean corpuscular hemoglobin concentration (MCHC), and platelet (PLT) count were not influenced by the addition of CS extract to the diet of pigs across all treatments (*p* > 0.05) (Supplementary Figure S 1). In addition, the results showed that supplementation with CS extract during PRRSV challenge did not significantly affect neutrophils, eosinophils, basophils, or monocytes (*p* > 0.05) on Day 7 or Day 14 post infection.

**Fig. 5:**
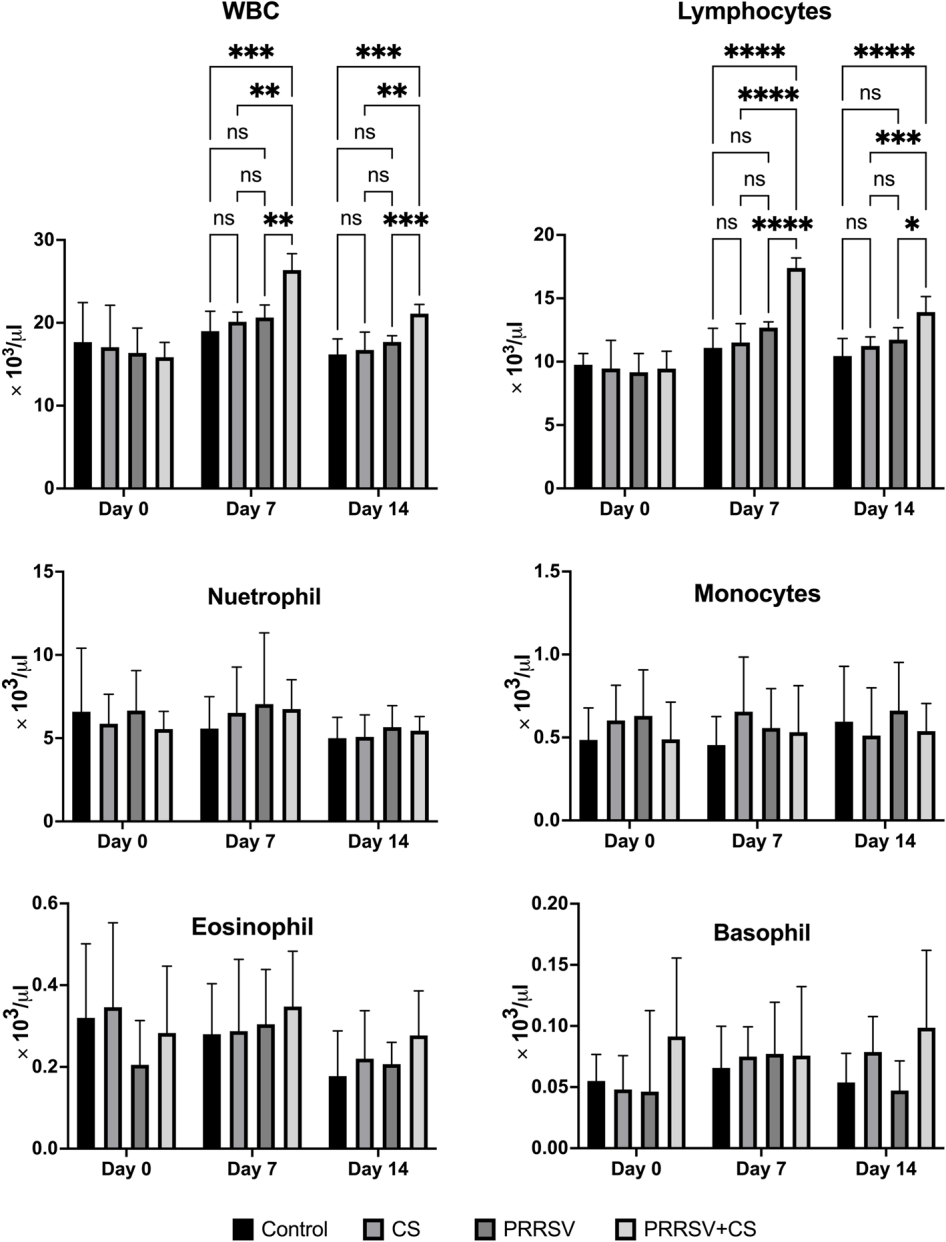
Effect of supplementation with CS extract on white blood cells following PRRSV challenge. The data are shown as the mean ± SD. The data are shown as the mean ± SD. The asterisks indicate significant differences (* *p* < 0.05; ** *p* < 0.01; *** *p* < 0.001; **** *p* < 0.0001) between groups

Compared to that in the PRRSV groups, the number of white blood cells (WBCs) increased most significantly in the CS + PRRSV group on Days 7 and 14 post infection (*p* < 0.0001); however, these increases were not statistically significant in the PRRSV groups (Fig. 5). Additionally, seven days postinfection, there was a notable increase in the total number of lymphocytes in the CS + PRRSV group. By Day 14, the number of lymphocytes in that group had decreased, but it was still significantly greater than those in the PRRSV groups (*p* < 0.0001).

### Gross pathology and lung histomorphology

**Fig. 6:**
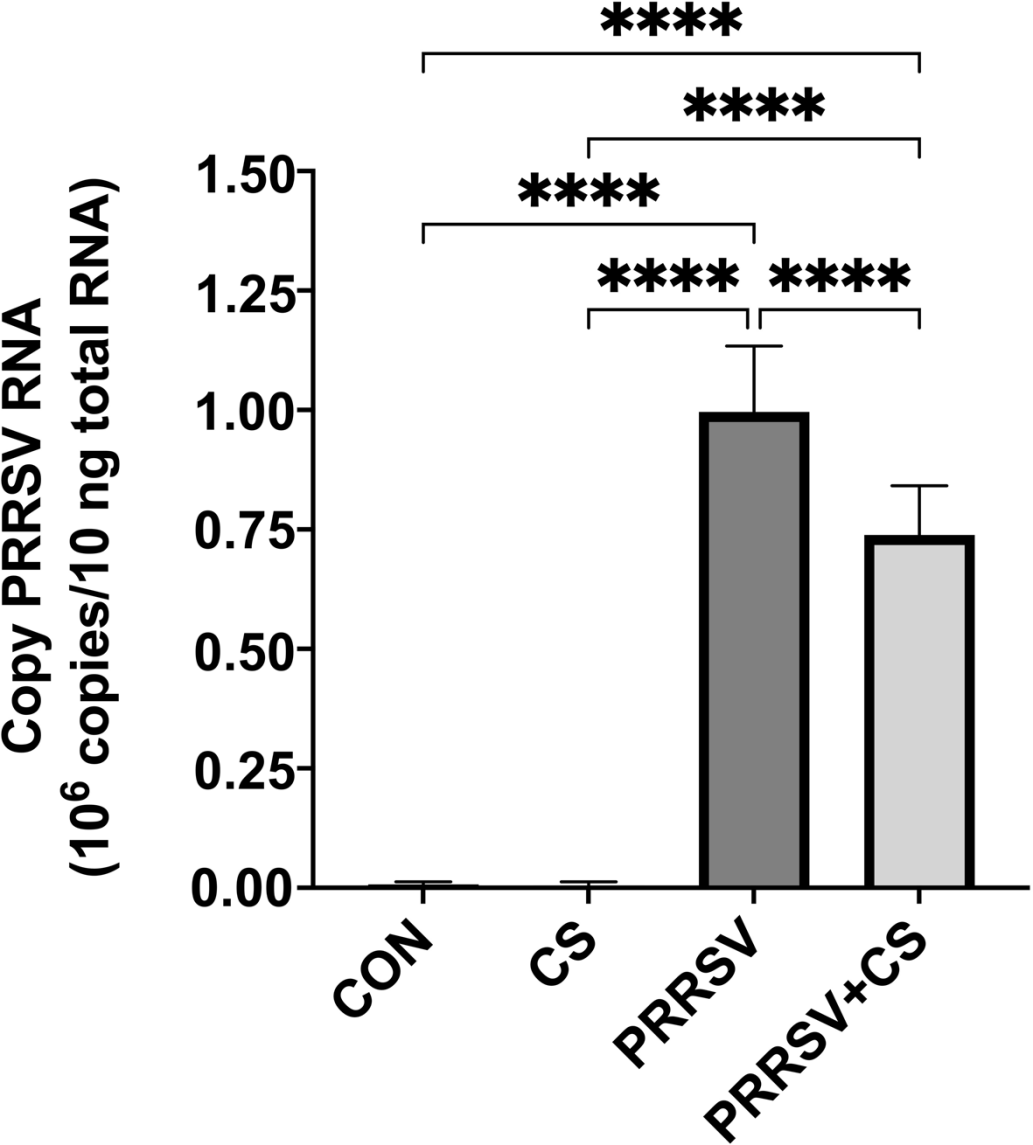
Effect of supplementation with CS extract on the number of PRRSV RNA copies in the lung following PRRSV challenge. The data are shown as the mean ± SD. The asterisk indicates significant differences (**** *p* < 0.0001) between groups

**Fig. 7:**
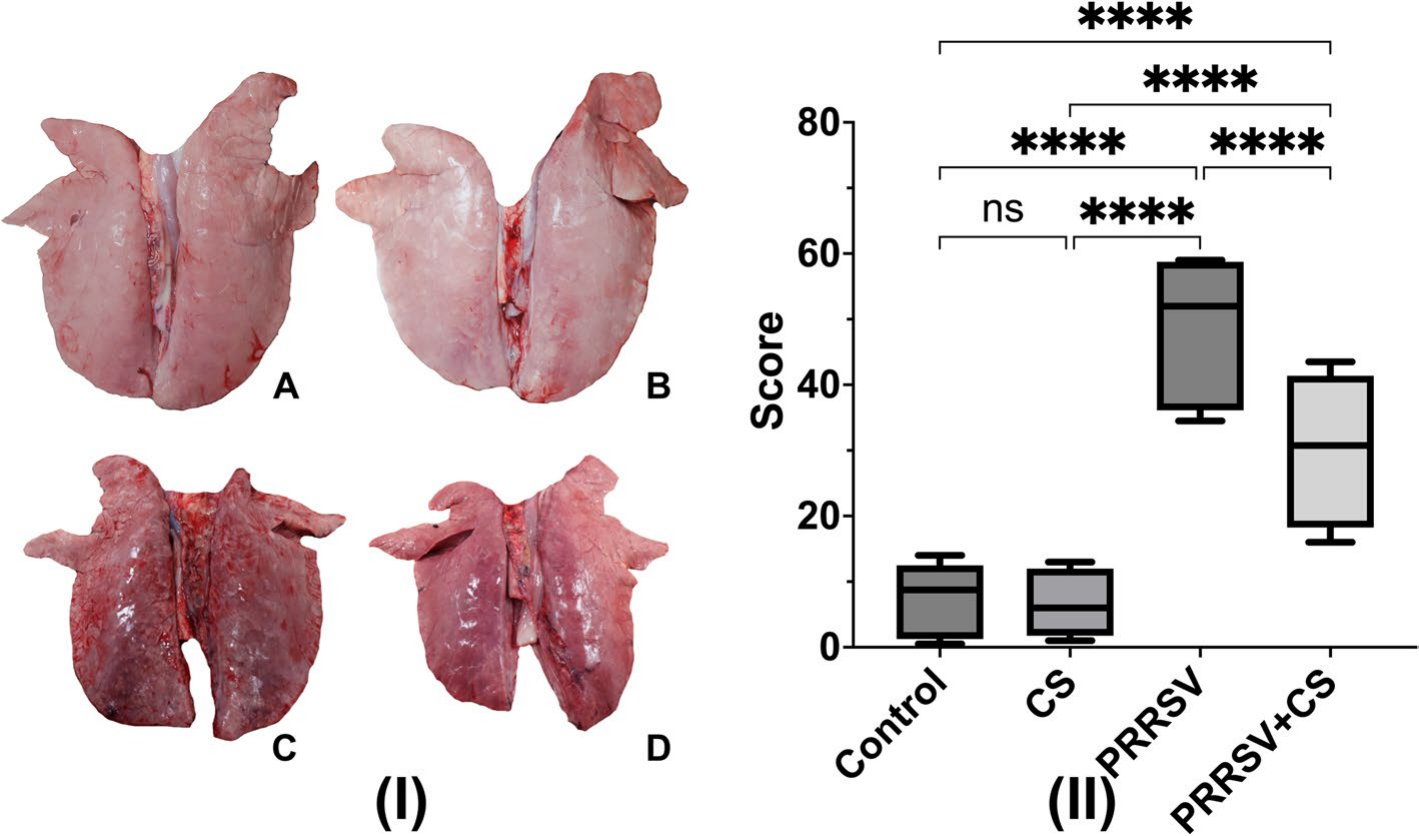
**I** Effect of CS extract supplementation on lung pathology in pigs. The letters indicate lung pathology in the CON (A), CS (B), PRRSV (C), and PRRSV + CS (D) groups. **II** Effect of CS extract supplementation on the average number of gross lung lesions in pigs. The average gross lung scores were evaluated as the percentage of lung tissue affected by pneumonia. The data are shown as the mean ± SD. The asterisk indicates significant differences (**** *p* < 0.0001) between groups

**Fig. 8:**
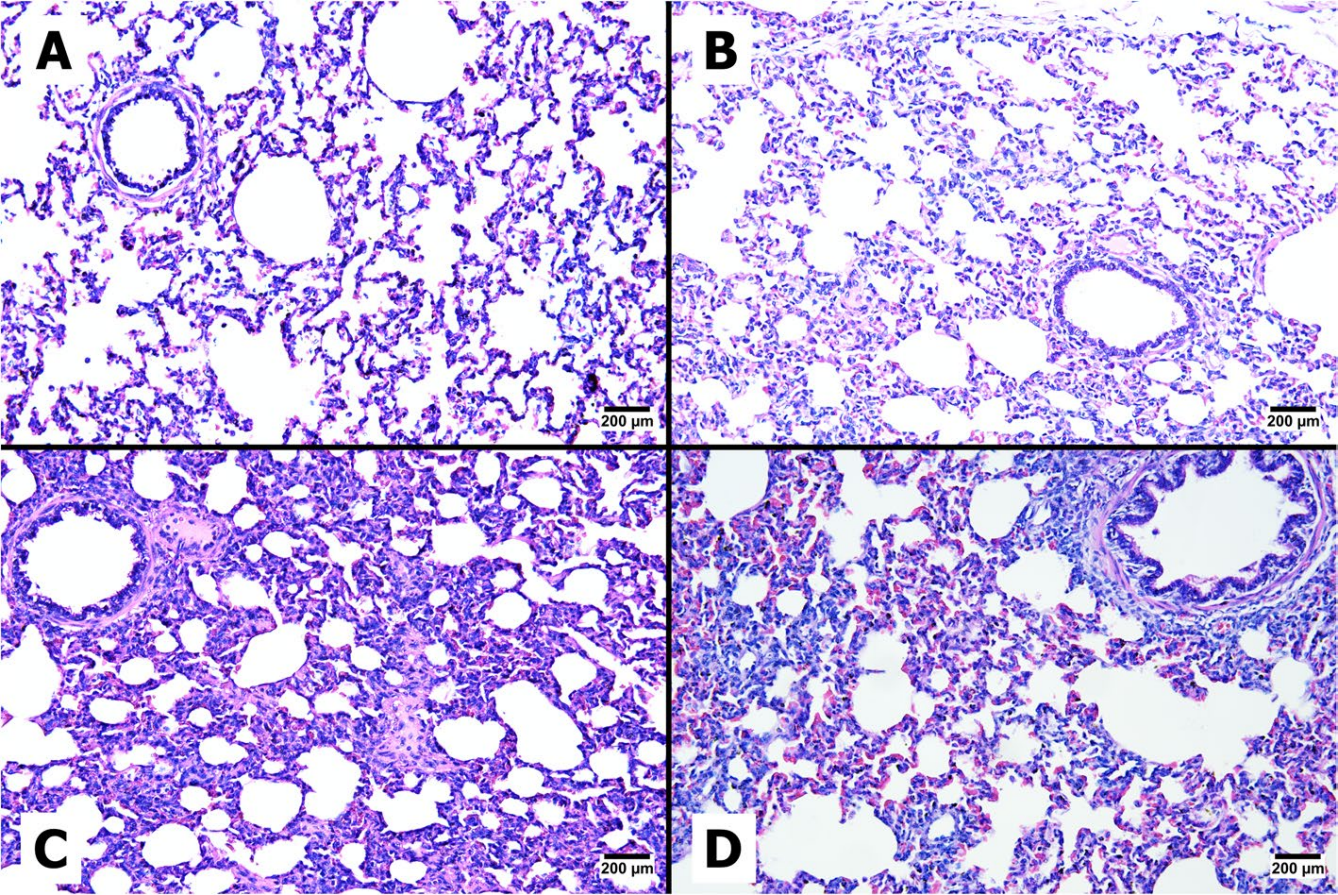
Effect of CS extract supplementation on lung histopathological lesions in CON (**A**), CS (**B**), PRRSV (**C**), and PRRSV + CS (**D**) pigs. Microscopic pictures at 200 × magnification

RT‒qPCR revealed that PRRSV copies were present in the alveolar tissues of both PRRSV-challenged groups (PRRSV and CS + PRRSV). However, the CS + PRRSV group had significantly fewer PRRSV copies than the PRRSV group (*p* < 0.001) (Fig. 6). The presence of viral copies induced lung lesions in the pigs. Supplementation with CS extract did not protect against lesions or damage to the lung after PRRSV infection. Compared with those from the sham group, the lungs from the PRRSV-challenged pigs exhibited gross lesions with hemorrhages and a dark color. The average lung lesion score was evaluated according to the percentage of lung tissue affected by pneumonia, necrosis, or hemorrhage, as shown in Fig. 7. The results showed that the PRRSV group had more lung lesions (46.56) than did the CS + PRRSV group (30.69) (*p* < 0.05). In addition, the histological analysis supported these findings. The lung samples from the sham groups lacked any characteristics that would suggest or indicate the presence of lung lesions. Multiple alveolar septal thickenings and macrophage infiltrations were characteristic of initial PRRSV-induced pneumonia in all of the challenged pigs (Fig. 8).

## Discussion

*Caesalpinia sappan* (CS) is a medicinal plant that has been widely used in Asian countries, including southern China, India, Myanmar, and Thailand. The plant is a rich source of flavonoids and phenolics [21]. Brazilin is the main flavonoid constituent found in CS heartwood [22]. As reported in our previous study, brazilin potentially prevents PRRSV infection by blocking the SRCR5 receptor on the CD163 protein [23]. This investigation revealed that 1 g of CS extract contained 93.58 mg of brazilin (data not shown). This dose corresponds to that in the study by Ruansit and Charerntantanakul [24], who administered 10 mg/kg quercetin in combination with a vaccination against highly pathogenic PRRSV-2 in pigs. The addition of 1 mg/kg CS was not detrimental to the pigs. Similar to those in the control group, the livers and kidneys of the CS-supplemented pigs had no lesions (Supplementary Figures S2 and S3). In agreement with the findings of Athinarayanana et al. [25], who reported that CS supplementation did not result in any evidence of toxicity or adverse reactions in organs (liver, kidneys, and other abdominal organs) of Wistar albino rats supplemented with large doses (100, 1,000, or 2,500 mg/kg body weight). Consequently, it is clear that CS extract is safe for use in pig supplements. During the study period, supplementation with CS extract had no significant effect on the productive performance (ADFI, ADG, WG, or FCR) of pigs with or without PRRSV infection (14 days). In the first two days following PRRSV infection, the appetites of the infected pigs diminished. Both the infected pigs that received the CS extract supplement and those that did not experienced decreases in appetite and feed consumption. The challenge with PRRSV was successful, as it induced an increase in rectal temperature and was associated with clinical signs in all the pigs, while those in the sham group did not display similar symptoms. An increase in body temperature was observed as the first sign of fever. There is mounting evidence that the increase in core body temperature of 1 to 4 °C that occurs during fever is associated with improved survival and the resolution of many infections [26]. The PRRSV-challenged pigs showed clinical signs such as lethargy and anorexia during the first two days post infection. In addition, dyspnea was also detected during the first two days post infection. Interestingly, we did not detect respiratory symptoms, such as coughing, in PRRSV-challenged pigs. The hypothesis is that the PRRSV strain in this study, VR2332 (North American genotype), may have less virulence in pigs than do novel strains, such as highly pathogenic PRRSV strains (HP-PRRSV). However, it is generally acknowledged that most infections caused by PRRSV are subclinical in chronically infected herds [27]. Consequently, the effects of the virus on the productive performance and clinical indications in this trial are unclear. The rectal temperature of the PRRSV-challenged pigs had increased on Day 4 post infection and remained higher than that in the sham pigs through the end of the experimental period. Conversely, compared with that in the sham group, the rectal temperature in the PRRSV-challenged pigs that received dietary supplementation with CS extract was significantly lower on Day 11 postinfection. Liu et al. [28] reported that PRRSV-challenged pigs fed plant extracts (capsicum oleoresin, botanical garlic, or turmeric oleoresin) had lower rectal temperatures than PRRSV-challenged pigs that were not fed the plant extracts. The authors also explained that the feeding of plant extracts might delay or shorten the occurrence of pyrexia in PRRSV-challenged pigs [28].

Supplementation of PRRSV-challenged pigs with the CS extract resulted in lower viral loads over time when compared with those in the PRRSV-challenged pigs that did not receive the CS extract. Thus, supplementation with CS extract might induce the production of cytokines, leading to the control of the viral titer and PRRSV copy number in the serum. This observation is in agreement with the findings of Pu et al. [29], who reported that PRRSV-challenged piglets that received *Hypericum perforatum* extract had significantly lower viral loads than did those in the control group. Liu et al. [28] explained that the reduction in viral load might indicate an antiviral effect of the plant extracts or may be related to the proliferation of B cells and T cells in the blood of PRRSV-challenged pigs. In any case, these findings may be related to the antibody titer in the serum after PRRSV infection. The addition of CS extract to PRRSV-challenged pigs (the CS + PRRSV group) significantly increased the S/P ratio of PRRSV-specific antibodies from Day 7 to Day 14 post infection compared to that in the PRRSV group. Generally, infection with PRRSV stimulates an antibody response by 7–9 days post infection [1]

PRRSV infection significantly impacted the immunity of the pigs, as indicated by an increase in the number of white blood cells on Days 7 and 14 following infection. After PRRSV infection, immunity is stimulated by an antibody response between 7 and 9 days post infection, but there is no evidence of protection against PRRSV infection [1]. The PRRSV-infected pigs that received CS extract supplementation had increased lymphocyte counts. Therefore, it is hypothesized that CS extract may increase the resistance of the adaptive immune system to PRRSV infection. In addition, the study revealed that the increase in lymphocytes correlated with the increase in WBC count. This study provides strong evidence that the number of lymphocytes in PRRSV-challenged pigs on Days 7 and 14 post infection was significantly greater than that in control pigs. Specifically, PRRSV-infected pigs that were supplemented with CS extract had higher lymphocyte counts than did the PRRSV group. In agreement with the findings of Rnochell et al. [30], who reported that PRRSV infection significantly increased the lymphocyte population on Days 7 and 14 post infection. Liu et al. [28] also showed that lymphocytes were suppressed in infected pigs after infection but increased on Day 14 post infection. After PRRSV infection, the number of NK cells, which are an innate lymphocyte subset that helps in the nonspecific clearance of any virus-infected cell from the body, increases [1]. Moreover, PRRSV infection had induced an increase in CD4 + T cells by 7 days postinfection that peaked on Day 14 postinfection [31]. CD4 + T cells play an important role in enhancing the adaptive immune system by activating other immune cells [28, 32]. Previously, it was found that CS extract can increase the population of CD4 + cells [33], indicating that CS extract supplementation stimulates the adaptive immune response in animals.

Clearly, the increased number of white blood cells and lymphocytes in the CS extract of PRRSV-challenged pigs (CS + PRRSV group) correlated with an increase in PRRSV-specific antibodies. The increase in PRRSV-specific antibodies may have been associated with the decrease in the blood viral load. The increase in the antibody titer suggested that the immune system recognized PRRSV and acted to eradicate the virus, resulting in a decrease in the serum viral load. The increase in lymphocytes in PRRSV-challenged pigs supplemented with CS extract was correlated with an increase in white blood cells, showing that CS extract supplementation augments immune system responses leading to elevated antibody titers following infection in pigs. In this study, we hypothesized that the addition of CS to the diet of PRRSV-infected pigs would help sustain the number of lymphocytes already present in the bloodstream. However, the underlying mechanism remains obscure, and further inquiry into these mechanisms will be conducted.

Supplementing PRRSV-challenged pigs with CS extract decreased the incidence of pulmonary lesions, which were more common in PRRSV-challenged pigs that did not receive CS extract supplementation. PRRSV infection triggers the secretion of interleukin (IL)-1β, an essential proinflammatory cytokine synthesized mainly by macrophages, monocytes, and dendritic cells that plays a critical role in coordinating the inflammatory and immune responses of the host system against invading pathogens [34, 35]. The increase in TNF-α and IL-1β levels during PRRSV infection may contribute to the increase in macroscopic lung lesions and fever in pigs in the challenged group because of the recruitment of additional immune cells to the site of infection [28, 36]. Therefore, supplementation with CS extract might protect against continuous inflammation. One of the primary compounds in CS is brazilin, which has various biological activities, including antioxidant [18, 37], antimicrobial [38, 39], antiviral [12, 40] and anti-inflammatory [41] activities. A recent report showed that brazilin suppressed the release of IL-1β and tumor necrosis factor-α (TNF-α) [19]. This might explain the reduced extent of the lesions in the lungs of PRRSV-challenged pigs supplemented with CS extract.

## Conclusions

In conclusion, the present study demonstrated that the addition of 1 mg/kg CS extract to pig diets decreased the virulence of PRRSV infection, resulting in a substantial change in immunity due to an increase in WBCs, lymphocytes, and antibody titers in the serum. Additionally, PRRSV-challenged pigs supplemented with CS extract had a lower viral load and fewer viral copies in the serum than PRRSV-challenged pigs that did not receive the supplemental CS extract. In addition, the use of CS extract reduced lung damage in pigs infected with PRRSV. This is the first report on the addition of CS extract to the feed of PRRSV-infected pigs with the aim of stimulating the production of antibodies. However, the mechanisms underlying the antiviral and immune system-inducing activities of CS extract in swine need further study.

## Materials and methods

### Samples and virus

Powdered CS heartwood was purchased from Chakkrawatherb Co., Ltd. (Bangkok, Thailand). PRRSV (VR2332 North American genotype) was propagated in MARC-145 cells (CRL-12231; American Tissue Collection Center (ATCC), Virginia, USA), and the virus was titrated using an immunoperoxidase monolayer assay (IPMA) and then stored at -80 °C. The virus titer was determined and expressed as TCID_50_ according to the Reed–Muench method [42].

### Animals and experimental design

A total of 32 weaned, 28-day-old ([Large White Landrace] Duroc) crossbred piglets from the CPF (Thailand) Public Company Limited were assigned to treatments in a completely randomized design. The piglets used in this study were verified to be PRRSV free. Pigs were housed in a biosafety level 2 facility chamber for 21 days [7 days before and 14 days after the PRRSV challenge (D0)]. As a feed additive for pigs, *C. sappan* extract was prepared and added to the diet. The treatments were in a 2 × 2 factorial arrangement (with or without PRRSV challenge and with or without plant extract supplementation). There were 8 replicates (piglets) per treatment. The treatments used were as follows: no challenge with PRRSV and no *C. sappan* extract in the diet (CON); no challenge with PRRSV supplementation with 1 mg/kg *C. sappan* extract in the diet (CS); challenge with PRRSV and no *C. sappan* extract in the diet (PRRSV); and challenge with PRRSV supplementation with 1 mg/kg *C. sappan* extract in the diet (CS + PRRSV). During the 14-day trial period, both groups of PRRSV-challenged and unchallenged pigs were housed with 2 piglets per pen in a biosafety level 2 facility with total isolation. On the initial day, each piglet in the PRRSV challenge groups was intranasally administered 2 mL of the US PRRSV strain at a median tissue culture infective dose (TCID_50_)/ml of 10^4^. The pigs in the unchallenged groups were given the same volume of normal saline (sham challenge). The piglets had unlimited access to feed and water. Table 2 shows the feeding formula used in accordance with the National Research Council (NRC) weaned pig nutrient requirements [43].

**Table 2:** Dietary ingredient composition

Items	Amount (%)
Corn	40.00
Broken rice	20.00
Fish meal	12.40
Soybean meal	12.00
Lactose	10.00
Whey	5.00
Dicalciumphosphate (DCP)	1.00
Vitamin premix^a^	0.25
Mineral premix^b^	0.35
Total	100
*Chemical composition*	
Metabolizable energy (Cal/kg)	3,450
Crude protein (%)	20.12
Ether extract (%)	6.02
Calcium (%)	0.80

^a^Vitamin premix (U or mg provided per kilogram of diet): vitamin A, 12,000U; vitamin D3, 4500U; vitamin E, 70U; vitamin K, 3.5 mg; vitamin B1, 3 mg; vitamin B2, 7.5 mg; vitamin B3, 30 mg; vitamin B5, 65 mg; vita- min B6, 4.3 mg; vitamin B9, 2 mg; vitamin B12, 0.025 mg; biotin, 0.3 mg; choline chloride, 800 mg

^b^Mineral premix (milligrams per kilogram of diet): 3,000 of from zinc oxide, 90 of Fe from ferrous sulfate, 20 of Mn from manganese oxide, 8 of Cu from copper sulfate, 0.35 of I from calcium iodide, and 0.30 of Se from sodium selenite

### Data and sample collection

The piglets and feed were weighed on the day before inoculation (Day 0) and on Days 7 and 14. The productive performance metrics, including average daily feed intake (ADFI), average daily gain (ADG), and feed conversion ratio (FCR), were measured for each interval from Day 0 to 7, Day 7 to 14, and Day 0 to 14 of the experimental period. The rectal temperature (RT) of each pig was measured before PRRSV inoculation (Day 0) and on Days 4, 7, 9, 11, and 14 postinfection. Additionally, the clinical signs were observed daily during the experimental period according to Ruansit and Charerntantanakul [24]. All pigs were euthanized on Day 14 postinfection. Prior to being euthanized, 1 ml of thiopental (sodium pentothal) was injected intravenously into the pigs' veins to induce anesthesia. The pigs were then euthanized by intravenous injection of saturated MgSO_4_ to induce cardiac arrest and death.

### Serum viremia and antibody titer

Blood samples were drawn from each pig's jugular vein on Days 0, 7, and 14 postinfection to collect serum. The serum samples were aliquoted and stored at -20 °C until viremia was detected via serum analysis and ELISA for PRRSV antibodies. The degree of viremia in the serum was determined using the IPMA virus titration method adapted from Pu et al. [44], and the results are expressed as TCID_50_ values. The ELISA antibody titer was determined using an IDEXX PRRS X3 Ab test commercial kit (IDEXX, Westbrook, ME, USA) according to the manufacturer’s instructions. All reagents required for the assay were provided with the kit, and the assay was conducted at room temperature. The optical density of each well was measured at 650 nm using a Bio-Rad 680 microplate reader. The presence or absence of the PRRSV antibody was determined by calculating the sample-to-positive (S/P) ratio. The samples were considered to be positive for PRRSV antibodies if the S/P ratio was greater than 0.4 [5].

### Quantitative real-time PCR (RT‒qPCR)

PRRSV RNA was isolated from serum and lung tissue with the PureLink™ RNA Micro Kit (Invitrogen) according to the manufacturer's guidelines. A Nanodrop spectrophotometer was used to quantify the RNA concentrations (Thermo Fisher Scientific). To synthesize cDNA from RNA, iScript Reverse Transcription Supermix was used for RT‒qPCR (Bio-Rad). Real-time PCR was performed using specific primers for the PRRSV ORF7 gene (forward: 5’ TCAICTGTGCCAGITGCTGG 3’ and reverse: 5’ AAATGIGGCTTCTCIGGITTTT 3’) with the US-PRRSV-specific probe FAM_US_rev (5’ TCCCGGTCCCTTGCCTCTGGA 3’, sense orientation) 5’-labeled with FAM [45]. Real-time PCR was performed on an ABI7500 instrument using a SensiFAST™ Probe NO-ROX Kit (Bioline) following the manufacturer’s instructions. For each experiment, a standard curve was generated using a serially diluted PRRSV standard of 10^3^–10^6^ TCID_50_/ml [10].

### Hematology

On Days 0, 7, and 14 post infection, blood samples were collected from each pig's jugular vein and placed in an EDTA tube to determine the total and differential blood cell counts. Differential leukocyte proportions and concentrations and red blood cells were analyzed according to the methods of Liu et al. [28]. The proportions of differential leukocytes and concentrations of red blood cells were analyzed on a multiparameter automated hematology analyzer calibrated for porcine blood.

### Gross pathology and histopathology of the lungs

The gross pig lung lesions were evaluated and graded using an established scoring system (100 points) and areas according to Halbur et al. [46]. In addition, lung tissue was collected and processed for histopathological examination. The samples were fixed in 10% formalin and stained with hematoxylin and eosin, as described previously [28].

### Statistical analysis

The statistical model included the effect of the PRRSV challenge, plant extract supplementation, and the interaction term as fixed effects. Specific contrasts were used to test comparisons between the control and the plant extract treatment groups collectively within each challenge condition. GraphPad Prism 9 (version 9.4.0; La Jolla, CA, USA) was utilized to conduct two-way analysis of variance (ANOVA) with Tukey's post hoc comparison. Viremia, antibody titer, and lung lesion data were analyzed within the challenged pigs only because no PRRSV-specific antibodies or viruses were detected in the sham-challenged pigs. Treatment differences were compared using the least squares means with a Tukey adjustment. The data are reported as the mean and standard deviation, and *p* < 0.05 was considered to indicate statistical significance. * *p* < 0.05; ** *p* < 0.01; *** *p* < 0.001; **** *p* < 0.0001.

### Supplementary Information

**Supplementary Material 1. **

**Supplementary Material 2. **

**Supplementary Material 3. **

## Data Availability

The datasets used and/or analysed during the current study are available from the corresponding author on reasonable request.

## Abbreviations

PRRS: Porcine reproductive and respiratory syndrome
PRRSV: Porcine reproductive and respiratory syndrome virus
CS: *Caesalpinia sappan*
ADFI: Average daily feed intake
WG: Weight gain
ADG: Average daily gain
FCR: Feed conversion ratio
TCID_50_: Median tissue culture infective dose
