# Supplementary figures and images for: Immune response enhancement by dietary supplementation with Caesalpinia sappan extract in weaned pigs challenged with porcine reproductive and respiratory syndrome virus
Source: BMC Vet Res. 2024 Mar 22;20:111. doi: 10.1186/s12917-024-03911-5 (PMC10958915; doi:10.1186/s12917-024-03911-5)

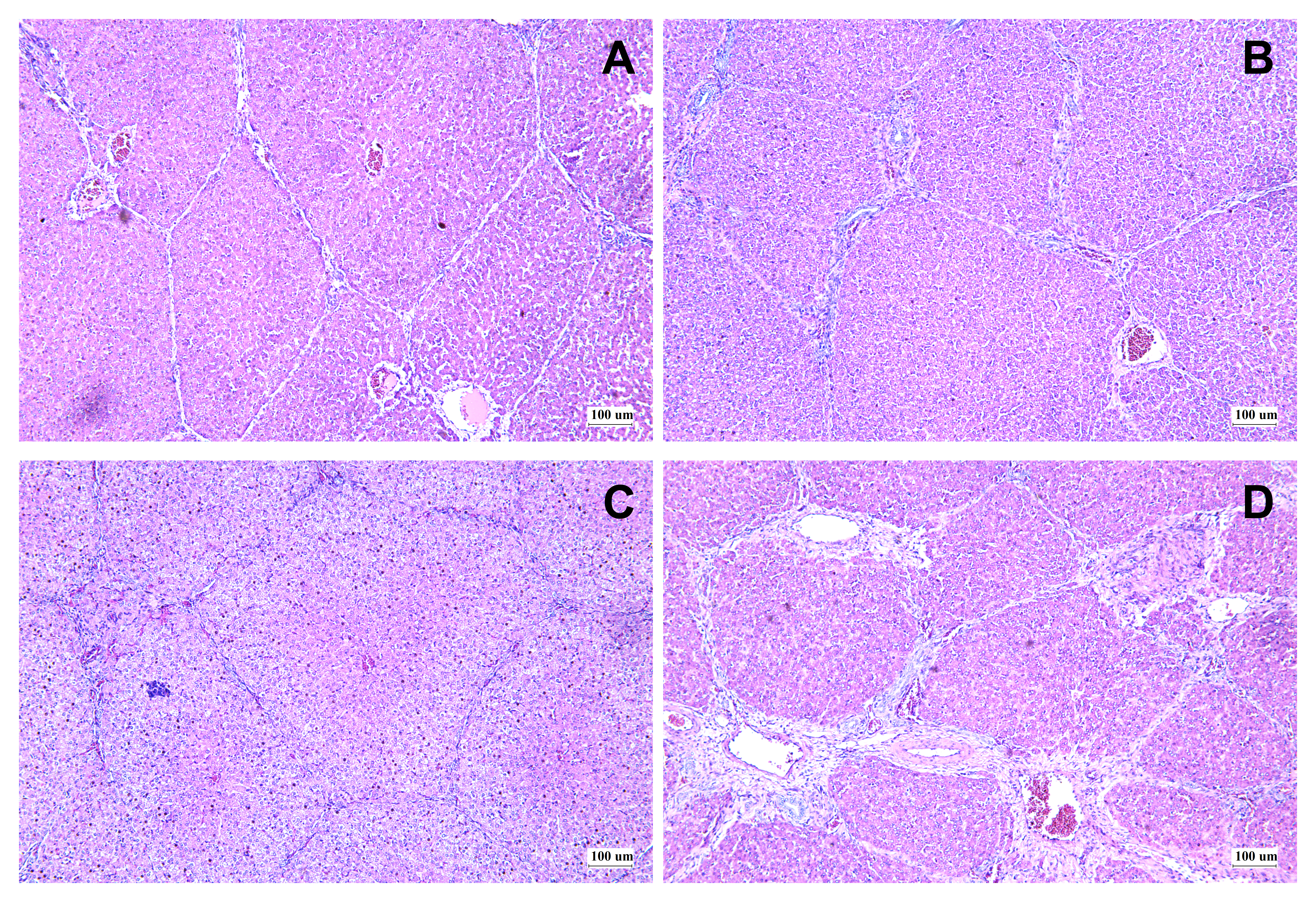

Supplement: Supplementary file 2 — Supplementary Material 2. [file 12917_2024_3911_MOESM2_ESM.png]

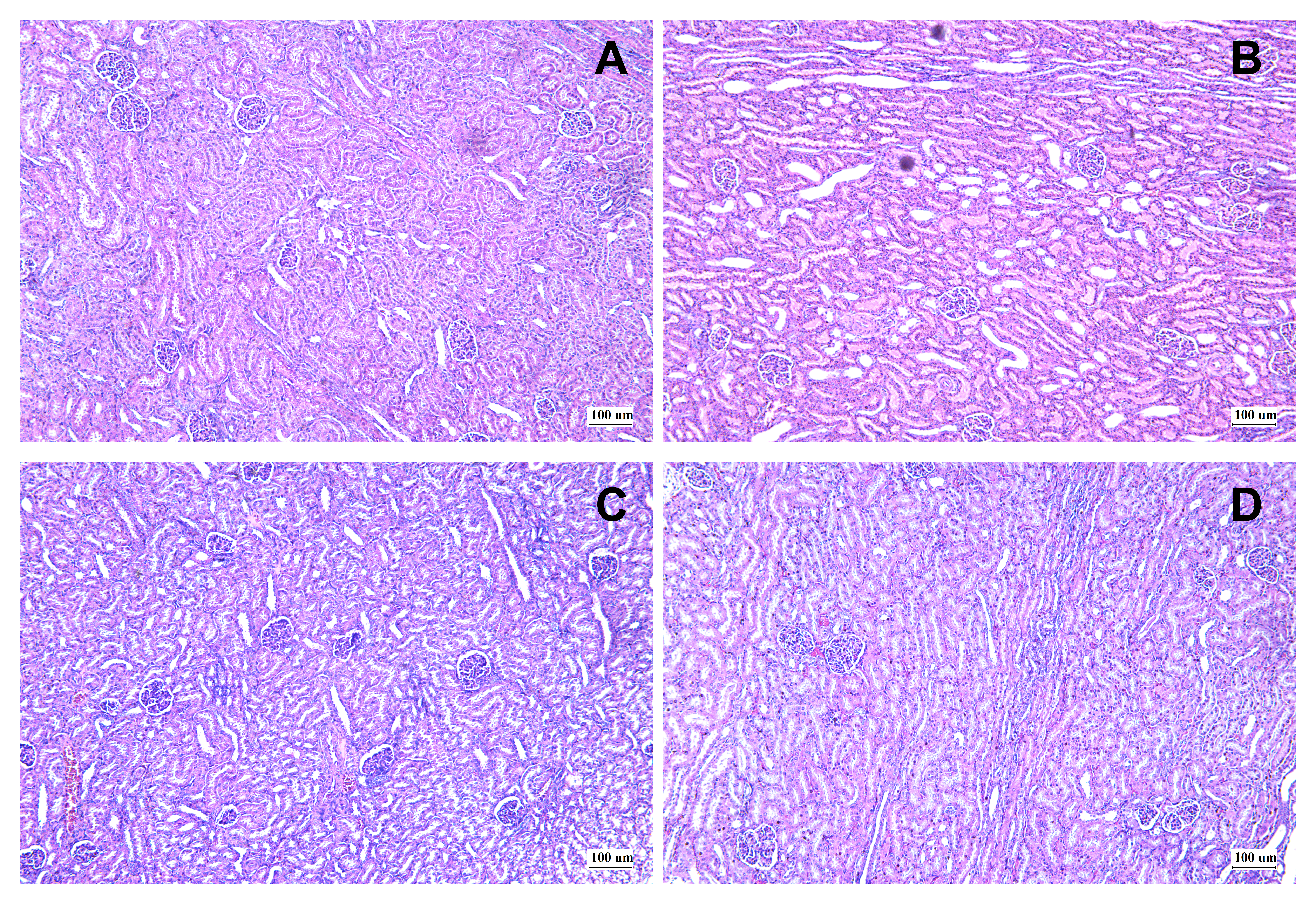

Supplement: Supplementary file 3 — Supplementary Material 3. [file 12917_2024_3911_MOESM3_ESM.png]
